# Health-related quality of life for adults living with hepatitis B in the United States: a qualitative assessment

**DOI:** 10.1186/s41687-021-00398-8

**Published:** 2021-11-10

**Authors:** Catherine Freeland, Rhea Racho, Maureen Kamischke, Kate Moraras, Evangeline Wang, Chari Cohen, Stuart Kendrick

**Affiliations:** 1 Hepatitis B Foundation, 3805 Old Easton Rd., Doylestown, PA 18902 USA; 2 FRCP GSK Research and Development, Stevenage, UK

**Keywords:** Hepatitis B Virus (HBV), Health related quality of life (HRQL), Patient reported outcomes (PRO), Viral hepatitis, Liver disease, Liver cancer, Patient experience, Antiviral therapy

## Abstract

**Background:**

In 2019, an estimated 296 million people were living with chronic hepatitis B virus (HBV) globally with approximately 2.4 million living within the United States. Those living with HBV require years if not decades of regular monitoring to prevent liver complications from occurring. The aim of this study was to explore the working conceptual framework of health related quality of life (HRQL) for those living with chronic HBV through qualitative analysis.

**Methods:**

Data were collected by in-depth telephone interviews conducted in 2019 using purposeful sampling as part of a disease understanding assessment on the HBV patient experience within the United States. A directed content analysis approach was utilized by creation of a codebook to guide the organization of data, codes were developed by review of the literature (a priori) and through line-by-line reading of a subsample of queries. All transcripts were analyzed by at least two members of the study team and intercoder reliability was assessed using Dedoose software.

**Findings:**

A sample of 19 individuals living with chronic HBV were included within this study. Themes identified from transcripts noted the significant overlap between the reported experience of HBV and constructs within the HRQL model. The psychological impact of chronic HBV on study participants’ HRQL overall was considerable and contributed to depression, anxiety, homelessness, drug use, and incarceration.

**Conclusion:**

Our analysis supports the hypothesis that HBV impacts HRQL and often negatively affects emotional health. Our findings suggest that it would be beneficial to include HRQL assessment in the medical management of HBV, so that interventions can focus on reducing the burden of disease and improving quality of life for those living with HBV.

**Supplementary Information:**

The online version contains supplementary material available at 10.1186/s41687-021-00398-8.

## Background

In 2019, an estimated 296 million people were living with chronic hepatitis B virus (HBV) globally with 2.4 million living within the United States [1, 2]. Left untreated, chronic HBV infection can lead to serious liver complications and premature death. In 2019, the World Health Organization (WHO) estimated that 820,000 deaths worldwide were attributed to HBV, mostly from cirrhosis and hepatocellular carcinoma (HCC, primary liver cancer) [1]. Globally, the WHO estimates that only 27 million people with HBV infection (10.5%) are aware of their infection, while 4.5 million of those diagnosed (16.7%) are on treatment [3]. Additionally, approximately 10–40% of those with HBV are eligible for treatment, meaning there is still a significant proportion of treatment eligible individuals who are not receiving it [3].

Those living with HBV require years if not decades of regular monitoring to prevent liver complications from occurring and improve HBV-related mortality [4]. There are antiviral therapies available that can effectively suppress HBV replication and decrease the risk of liver failure and liver cancer for those that are treatment eligible [5–7]. According to current treatment guidelines, only a small percentage of people with chronic HBV meet eligibility criteria for antiviral therapy, even though those with chronic HBV who do not meet criteria for treatment can still be at increased risk for developing HCC [8, 9]. Additionally, current antiviral therapy needs to be taken for many years and generally, those who start antiviral therapy for HBV suppression can expect to be on treatment for life [6]. Although treatment with antivirals significantly reduces the risk of liver cancer, it cannot eliminate risk, or reduce it to the level of a non-infected individual [9].

Patient reported outcome (PRO) measures are defined by the U.S. Food and Drug Administration (FDA) as “any report of the status of a patient’s health condition that comes directly from the patient without interpretation of the patient’s response by a clinician or anyone else” [10]. Common PRO indicators encourage the assessment of patient reported symptoms as well as health-related quality of life (HRQL) [11]. HRQL is an essential component in evaluating health and is a multi-dimensional construct including psychological, physical, and social functioning, in addition to other dimensions that are affected by a health condition [12, 13]. Previous research has demonstrated that HRQL in those living with HBV decreases as the disease progresses into more severe stages [14]. However, there has been limited research published on HRQL for those with chronic HBV, despite it being the most common viral infection in the world. Published literature has examined HBV using metrics for reporting HRQL (EQ5D self-reported questionnaire); however, these metrics might not be specific enough for those affected by HBV and lack qualitative research assessments to explore the concept of HRQL within the disease state. In 2007, one HRQL metric was developed for HBV that was created prior to the more recent antiviral therapies approved by the FDA in 2008 and 2016 [14–16]. The current and only HRQL framework for HBV is only mathematically described with limited definitions of the HRQL dimensions limiting its use in practice [16].

The aim of this study was to qualitatively explore the working conceptual framework of HRQL for those living with chronic HBV within the United States. Data gathered from interviews of individuals living with HBV were evaluated to assess quality of life implications of HBV and current experiences on HBV treatment.

## Methods

### Data collection

Data were collected as part of a disease and treatment understanding assessment conducted on behalf of GSK by an independent research group with the objective to understand the HBV patient experience including disease impact and areas of need. Individuals living with HBV were asked a series of questions regarding their experiences living with hepatitis B including their diagnosis, symptoms, current treatment experiences, concerns, and future treatment preferences through qualitative interviews lasting approximately 60 min in length. Recruitment utilized a patient panel conducted in the United States, identifying participants who were then approached by their healthcare providers. Eligible participants were identified and approached by their healthcare providers who were members in a research panel. Individuals above the age of 18 living in the United States, and with current chronic HBV diagnosis confirmed by the healthcare provider at the time of enrollment were invited to participate. The sample size was estimated to be sufficient to achieve data saturation and an iterative sampling strategy was not employed. Data were collected by in-depth telephone interviews conducted between April to November 2019 by trained researchers using a structured discussion guide (Additional file 1: Appendix). Interviews were transcribed verbatim. Non-English transcripts were translated into English, and back-translation was used to ensure meanings were adequately captured in translation. Analysis was approved by the Heartland Institutional Review Board. No identifiable information was included during data extraction. Participants gave informed consent to have the anonymized transcripts shared, analyzed, and published with the purpose of helping other patients, healthcare providers, caregivers, or others who have a role in the treatment of people with HBV understand their experiences, perceptions, and needs related to HBV.

### Data analysis

A directed content analysis approach was utilized by creation of a codebook to guide the organization of data, and codes were developed by review of the literature (a priori) and through line-by-line reading of a subsample of queries [17]. Each code was given a specific definition to ensure coding accuracy and to improve intercoder reliability [18]. The codebook is provided in Additional file 1: Appendix. All data transcripts (N = 19) were independently double coded by two members of the research team (CF, RR, MK, CC, KM) to ensure coding accuracy. Inter-coder reliability (ICR) was assessed to identify coding discrepancies, and the analysis team met throughout the coding process to discuss and resolve discrepancies in coding. After coding was complete, the team reviewed the coding and organized findings into thematic categories guided by HRQL constructs. Data analysis was facilitated using Dedoose, a software program that facilitates organization of qualitative data.

## Results

A sample of 19 individuals living with HBV were interviewed who identified as living with chronic HBV in the United States. Participants self-identified as African American (21%), White (42%), Asian (31%), and Hispanic/Latino (5%). The sample were 53% male, currently participating in antiviral treatment for HBV (79%), reported a co-infection with HIV, HCV, or HDV (32%), and older than 35 years of age (95%) (Table 1). Analysis of transcripts revealed significant overlap in the HRQL model and the experiences of those living with HBV. Using the HRQL model, we will outline constructs providing detailed quotes from those living with HBV.

**Table 1:** Demographic characteristics for participant sample of those living with HBV in the United States

	Number	Percent (%)
Participants	19	100
Age		
35 or under	1	5
36–45	4	21
46–55	4	21
56–65	5	26
> 65	5	26
Male	10	53
Currently treated	15	79
Physician type		
Primary care	7	37
Hepatology	4	21
Gastroenterology	4	21
Infectious disease	4	21
Cirrhosis	1	5
Coinfection		
HIV	4	21
HCV	1	5
HDV	1	5
Education and employment		
Unemployed	1	5
College/employed or retired	12	63
College/unable to work	1	5
High school/employed or retired	3	16
High school/unemployed	0	0
High school/unable to work	2	11

*HIV* human immunodeficiency virus, *HCV* hepatitis C virus, *HDV* hepatitis delta virus

### Psychological well-being

Those living with HBV described significant psychological implications after diagnosis. One individual shared, “I shut down. I didn't talk to anyone.” Another described, “I didn't feel anything…I was numb…I didn't want to feel any pain so I tried to stay high. I ended up being homeless… I ended up losing everything and I was at my rock bottom. I lost myself. I lost my dignity. I was gone. I just gave up like a mission to suicide.” A participant shared, “Everything changed, and I mean everything…my whole life. At first, I started getting depressed and I went through a lot of depression, and I went drug use, prison again.” Further, it was explained, “When I saw that first doctor and she didn't inform me of anything much and I didn't pursue it. You know, sometimes we do things by not dealing with them, by denial.” Others described coping mechanisms after diagnosis, “My story has been to block it out. I don’t worry about the actual disease or any affects what I pretty much do is just take the medication and just pray for the best.” One individual shared, “I'm trying to push onward and not let the situation with hep B like be all encompassing.” Similarly a participant stated, “Sometimes I can get depressed if I do think about all the things I’ve been going through with my health. I do get depressed at times, but I try to just go about my day and my life as normal as I can.”

#### Fear and anxiety

The most commonly reported finding within data related the fear and anxiety after an HBV diagnosis. Those living with HBV described the initial fear but have since coped with their diagnosis stating, “In the beginning it was pretty stressful but now, today to see me living a life such as the one that I’m living today is not so much as hectic.” Another person shared, “At the time it was diagnosed at that time we were all scared. We were all considering what will happen in the future.” People shared concerns about overall health and longevity after an HBV diagnosis. One individual shared, “I am worried about having bigger complications down the line.” Another echoed this saying, “Yes, I am scared like if the virus level increases I have to take medication but that scares me.” One individual stated, “The fear I have is like if the viral level is high and if it is not curable what will be my condition? Who will take care of me? Who will take care of my family? And what condition I will be in.” Participants shared other fears of liver cancer compared to liver damage or cirrhosis, “I’m not necessarily worried about cirrhosis that much because I think cirrhosis, it does happen but I’m worried about liver cancer.” One individual described lifestyle modifications to improve vitality, “I actually stopped drinking alcohol three years ago after reading because I don’t want to have liver cancer.” One patient also described, “I was like “oh s**t now I am older than 40 so I’m at a higher risk now” and I’ve also read about how serious liver cancer is and how basically it’s not curable. It’s one of the harshest cancers out there and usually detected when it’s too late…That’s why it’s like a time bomb. You just don’t know when it’s going to explode.” Others have shared coping mechanisms, “[I] take control and create order out of chaos… So, I’ve spent probably the last eight years reading on the disease, reading the latest studies, proactively reaching out to my doctors, and proactively looking for clinical trials and that sort of thing. So, I feel like I’m taking all these different things as information and kind of turning it down to what I need.”

### Physical

In total, 8 of the 19 participants reported no signs or physical symptoms associated with their hepatitis B infection and described, “There were no symptoms, nothing.” While much of the literature echoes this sentiment, that often hepatitis B is asymptomatic, the majority of individuals participating in this study were diagnosed as a result of their initial symptoms of fatigue, nausea, pain in the abdominal region and other flu like symptoms. One individual describes experiencing jaundice, “I had my eyes turn yellow and I got real tired and I just wasn’t feeling good.” A participant shared their symptoms of, “nausea, vomiting, pain on my right abdomen and headaches.” Another describes the significant impact of the symptoms on their daily life, “I did get really, really sick from it. I learned to live with it, a little bit. I was really tired a lot and it changed my whole life.” A patient with known cirrhosis mentioned experiencing symptoms in this stomach sharing, “Just my stomach is big and bloated and I know that’s a direct cause of that.” Similarly, an individual described, “pain like bloated, below the ribs.”

### Social functioning

#### Concerns about transmission and disclosure

Those living with HBV shared significant concerns of transmission to loved ones. One person shared, “They’re always afraid that you know, don’t touch grandma, don’t touch her cuts because they don’t want their kids getting sick and it’s kind of embarrassing but it’s understandable.” Another individual described, “I think part of it literally is the fact that I don’t want to deal with having to tell a significant other because I just don’t want to have to get into that conversation.” Others discuss similar examples of caution related to relationships. A person explained, “It’s a little bit of a barrier just because I think maybe I’m my own worst enemy and I’m not letting myself get involved with anybody. After I’ve learned everything about it it’s not really something that another person needs to really worry too much about because people get vaccinated for it now.” Similarly, an individual echoed, “I’m always going to be concerned about making someone else sick. I think even when you tell me I’m cured.” While most people expressed fear and hesitation around disclosure, there were a couple individuals that were more comfortable sharing their HBV status and stated, “I tell everyone. Anybody that has anything to do with me. Like, anybody that comes in contact or touch me, I tell.” Another individual similarly said, “Yes, I don't hide it, I'm not ashamed of it, I don't think it's taboo. Yes, a lot of people know.”

#### Stigma

A common experience related to those with HBV is social isolation as well as the experience of internal and external stigma. One individual described their experience with internal stigma, “There's nobody that's made me feel that way. I've felt that way [guilt, shame] just on my own.” Others shared similar sentiments describing the fear associated with disclosing one's HBV status. A participant described, “Just the social aspect of it, being afraid to tell people just that kind of stigma I wish that was gone.” Similarly, another individual shared, “I don’t have any friends or family members that have this problem and I’m not going to go around and advertise it, I can’t. It’s part of the stigma. Nobody knows about what I have except my own family or wife.” Another person affirmed, “[HBV] is not something you tell. Only your family should know because they live with you.” A father also discussed the external stigma with his daughter starting school sharing, “When my daughter was born and she was like three or four years old and went to school and I had to tell them. It was just horrible. Telling them that she [has] hepatitis was like telling them that she had the plague, they said, “well, we’re not going to have her come to school until we absolutely have to have her here” and it was just horrible. And it was just… there were times when she cried, she couldn’t play with some kids and it broke my heart.” Others described embarrassment and feeling like a “leper” or suggested that people with HBV were involved in “doing drugs or needles, or too many tattoos, or promiscuity." One individual shared, “I do feel the stigma in the sense that I don’t want to share it with people because I think a lot of people think if you have hepatitis it’s from IV drug use.” Another individual shared, “Sometimes I feel like I’m a different person than everybody else, I’m not as equal to everybody else or I’m less than a person since I got this but in my mind I know that’s just me. Just because I have a disease doesn’t make me a bad person.”

### Current treatment experience

When asked about current HBV antiviral treatments, participants reported a variety of experiences including, “contentment,” “acceptance,” and “burden.” One person described, “It’s pretty normal. I take a pill every morning and that’s it, every six months I get checked by a GI doctor, I get an ultrasound, I get labs drawn and there’s always a period of “Oh let’s hope everything is good,” and then go back to normal life. So that’s been my routine now for seven years, eight years.” A participant explained, “It hasn’t really affected me too much in that sense, in my day-to-day life because I don’t really do anything differently other than remember every morning it’s automatic, I just wake up and take a pill and go about my day.” One described the burden of HBV treatment, “Like I said, at first, it kind of depressed me, because here we go, another medication to take for the rest of my life, I already have to take HIV pills for the rest of my life but, like I said, I take several already, so it’s like, all right, add one more to the mix.” One individual described, “Yes, it's worked just fine. One of the hard things with taking it, is that you have to take it two hours before you eat and … Two hours before and two hours after, so, in other words, you can't eat for four hours. So, trying to figure out a good time to take it. It's confusing to me too.” Others commonly described treatment as “It's become part of my life.” The longevity of treatment was a significant patient concern, “Once a day. Well, that’s a little bit of a hassle too, not necessarily taking the medication but when I started on this medication, she told me “Once you start on this you have to take it for the rest of your life.”

Others described fear associated with starting treatment. One person shared, “Yes, she [doctor] wants me to go on some medicine and I’m like, I told her, I’ve had it this long, I’m not going to be around a whole lot longer anyways, so I don’t need it and she goes’ “no (de-identified), you need this. This medicine is good now. It’s not like it was years ago and made people sick… So, I just say, well I’ve learned to live with all these years, you know what, what’s going to be a few more years.” Another expressed contentment with current treatment and shared, “I'm happy with the treatment right now, so that’s my… the DNA virus is undetected.”

## Discussion

This qualitative study highlights the lived experiences of a sample of 19 individuals living with chronic HBV infection in the United States. Overall, among study participants, HBV directly impacted HRQL in each construct of psychological, social functioning and physical health. While HBV is frequently reported within literature as being asymptomatic, many study participants reported on the physical symptoms they experienced because of HBV. However, the data suggest that living with chronic HBV had a greater overall impact on the psychological and social functioning of participants than on their physical well-being. This can be seen in the interview data, as participants spent more time discussing the psychological and social functioning impacts and were also more descriptive in those interview question responses. The psychological impact of chronic HBV on study participants’ HRQL overall was considerable and contributed to depression, anxiety, homelessness, drug use, and incarceration. Every participant interviewed shared how their HBV diagnosis impacted their lives. For some it had a more significant impact and “changed their whole life” while others suggested their diagnosis resulted in lifestyle modification. From data it is clear that the impact of HBV can be substantial in the form avoiding alcohol or tobacco, or leading to feelings of self-stigmatization, numbness, depression, denial, and succumbing to substance use. Within the literature, there has been limited research on an individual’s reaction to HBV diagnosis [19]. One qualitative study in Iran found that after a chronic HBV diagnosis, participants faced emotional challenges including denial, anger and aggression, which they needed to control and manage over time [19]. With limited published data, one can look at similar disease states like HIV where there has been extensive research. One study describes one’s HIV diagnosis as complex, multi-faceted, and evolving over time [20]. This same study found that individuals often were unable to accept or integrate the knowledge of their diagnosis into their sense of self, sometimes for as long as a decade or more and describe this as a sense of denial [20]. These findings within HIV might be applicable to HBV based on our findings, but more research is needed to explore the rationale behind various reactions to an HBV diagnosis. Ideally, this research could contribute to a better understanding and lead to effective interventions to address HRQL challenges associated with HBV diagnosis.

Data from participants demonstrate how the fear of transmission influences behavior of both the individual living with HBV and those around them. These findings are validated consistently throughout literature [16, 21–24]. This includes feelings of guilt and shame associated with diagnosis which is a significant psychological impact that can lead to self-stigmatization. This self-stigmatization can contribute to poorer health outcomes and has origins within an overall lack of knowledge and awareness of HBV in the broader population [21]. Similarly, those living with HBV described the fear of disclosing one’s status and reported avoiding relationships due to their HBV. This fear of disclosure has been reported in qualitative research and associated with various forms of rejection [25, 26]. Overall, our findings demonstrate that HBV diagnosis can lead to isolation, both of self and by community, feelings of guilt, shame, and avoiding relationships to evade disclosure which has a significant impact on overall HRQL for those living with HBV.

Within this study, those interviewed expressed the constant fear of the progression to liver cancer and loss of vitality after diagnosis. One participant described their HBV and the fear of liver cancer as a “ticking time bomb” because it most often has poor prognosis and outcomes. Additionally, the “burden” of daily medication to reduce the risk of liver cancer and control HBV is daunting for many individuals but has become a way of life. In previous literature on patient reported outcomes for those with chronic HBV, poorer HRQL is consistently related to advanced liver disease [21]. Our study offers evidence that HRQL is negatively impacted regardless of liver disease status, on both an emotional and psychological level, as people face worry, fear, stigma, and lifestyle burden. Ultimately, it is clear that HBV has a significant impact on HRQL, affecting psychological well-being and feelings of vitality. Future research should continue to explore and expand upon the HRQL impact on chronic HBV patients to fully understand its impact.

From data, overarching themes identified for individuals living with HBV significantly overlap with the HRQL model. HRQL is a multi-dimensional concept related to physical, mental, emotional, and social functioning [27]. Our analysis supports the claim that HBV impacts health-related quality of life and often negatively affects emotional and mental health. For those with HBV, without symptoms, the possibility of progression to liver cancer and the lifelong nature of infection leads to the perception of having severe disease regardless of disease state [28, 29]. Previous studies have linked the infectious nature of HBV, inadequate knowledge about transmission modes, and anxiety about transmissibility of the virus to social isolation for those infected [29]. This isolation, as well as stigma and discrimination might be reasons for the evident psychological burden associated with HBV [29]. Based on our research, the HRQL scale created by Spiegel et al. captures HRQL related to HBV based on factors identified within this study [16]. Future research should further quantify HRQL within patient populations in the United States building off previous/existing research [15, 16]. The HRQL research for HBV should be expanded upon to a broader population of chronic HBV patients, to better clarify the impact and support the development of appropriate interventions to mitigate the negative aspects of living with HBV. Assessing and improving HRQL has played an important role in the management of other chronic conditions, and we suggest that professional medical societies consider including the impact of HRQL in the clinical management of HBV into the future.

### Limitations

This study in its qualitative nature has several limitations that should be pointed out. Although this research was able to recruit individuals living with HBV, the study sample may not be representative of all individuals living with HBV and is only representative of 19 individuals’ experiences living with HBV within the United States. The research questionnaire (Additional file 1: Appendix) while it encompasses significant aspects of HRQL was not solely for the purpose of assessing HRQL concepts. Future research should continue to explore HRQL specifically for those with HBV in a qualitative manner. There may be uncommon experiences not captured in the data which were nevertheless significant HRQL impacts for a minority of people living with HBV. Additionally, it is important to note that this sample consists mostly of individuals over the age of 35, many with co-infections and on treatment. It is important to explore other perspectives of individuals living with hepatitis B who might not be on treatment, those not co-infected with other infectious disease or those under the age of 35. Because this study questions focus on hepatitis B alone and not the interaction between other infections, we still believe that the findings accurately depict the experience of hepatitis B, and for some individuals that experience involves co-infection. Additionally, geographic information from participants was not collected as part of the questionnaire and regional or geographic differences within communities experiencing HBV should be explored in future work.

## Conclusion

This qualitative study highlights the lived experiences of a sample of 19 individuals living with chronic HBV infection in the US. Every participant interviewed shared how their HBV diagnosis impacted their lives. Overall, the diagnosis and lived experience with HBV had a clear impact on the constructs outlined within the HRQL model. From our sample, the psychological impact of chronic HBV on an individual’s HRQL overall is considerable, starting at diagnosis, and can contribute to depression, anxiety, and self-isolation. The emotional impact is exemplified by fear of disclosure and transmission to others, and fear of progression to liver cancer and loss of vitality. Together, the psychological and emotional impact, along with HBV-related stigma, can lead to isolation and result in a range of coping behaviors – from healthy lifestyle modifications to denial to drug use. This research demonstrates the continued need to explore and quantify HRQL for those living with HBV and the importance of PRO measures for evaluating the patient experience. An ideal HRQL metric should include specific HBV domains of psychological well-being, fear and anxiety, concerns of HBV transmission and disclosure, and stigma. Our findings suggest that it would be beneficial to include HRQL assessment in the medical management of HBV, so that interventions can focus on reducing the burden of disease and improving quality of life for those living with HBV.

## Supplementary Information

**Additional file 1: Appendix.** Structured interview guide for individuals living with hepatitis B on their lived experience.

## Data Availability

Transcripts for data are available on request to corresponding author. Code book and methods are made publicly available within Additional file 1: appendix.
