# Supplementary material for: Health-related quality of life for adults living with hepatitis B in the United States: a qualitative assessment
Source: J Patient Rep Outcomes. 2021 Nov 10;5:121. doi: 10.1186/s41687-021-00398-8 (PMC8581088; doi:10.1186/s41687-021-00398-8)
Supplement: Supplementary file 1 — Additional file 1: Appendix. Structured interview guide for individuals living with hepatitis B on their lived experience. [file 41687_2021_398_MOESM1_ESM.docx]

**Additional file 1: Appendix:**

**List of Abbreviations**

US (United States)

Hepatitis B Virus (HBV)

Hepatocellular Carcinoma (HCC)

World Health Organization (WHO)

Patient Reported Outcomes (PRO)

Health Related Quality of Life (HRQL)

Hepatitis C Virus (HCV)

Hepatitis Delta Virus (HDV)

Human Immunodeficiency Virus (HIV)

**Codebook used to guide qualitative data analysis.**

| **Code** | **Definition** | **When not to use** | **Number of References within Transcripts** |
| --- | --- | --- | --- |
| **Diagnosis** | Use when individual shares or asks questions about hepatitis B diagnostics or describes their diagnosis with HBV(e.g. provides lab report, or lists out lab results, acute vs. chronic) | Do not use when individual is discussing cure, prevention (vaccine), transmission. | 181 |
| **Transmission** | Use when participant discusses exposure or transmission of infection to others (i.e.- sex, needle stick, mother to child, blood exposure), or a general question/comment about how hepatitis B/D is transmitted | Do not use when vaccine is mentioned, when test results are listed. | 85 |
| **Vaccination/Prevention** | Use when there is anything to do with vaccination or hepatitis B/D prevention or prevention of transmission from occurring, | Do not use when discussing test results, or anti-vaccine discussion is present, or if transmission or exposure is discussed | 18 |
| **Healthy lifestyle** | Use when discussing exercise or eating healthily while living with hepatitis B & D, any discussions related to living with hepatitis B – including questions about what to avoid, maintaining a healthy lifestyle, healthy liver, things to avoid. | Do not use when discussion of herbal supplements or alternative therapies are mentioned. | 95 |
| **Discrimination** | Use when any type of institutional discrimination is involved in conversation, can be related to employment, visa, school etc. | Do not use when referring to social stigma or being treated differently by individuals. | 8 |
| **Symptoms** | Use when symptoms of hepatitis B and D are discussed (jaundice, fever, flu like symptoms, itching, side pain, fatigue) | Do not use when talking about medication side effects, and other illnesses symptoms, general pains, pain or symptoms not related to hepatitis B. | 100 |
| **Liver Cancer & Liver Damage** | Use when liver cancer and/or cirrhosis, fibrosis (scarring of the liver, fibro-scan (F4)) is discussed related to hepatitis B and D | Do not use when cure is discussed | 49 |
| **Cure or Clearing the Virus** | Use when discussion of hepatitis B/D cure is discussed or possibility of clearing the virus naturally | Do not use when referring to treatment options or access to treatment/medication | 81 |
| **Stigma** | Use when discussion of being treated differently by peers/individuals due to hepatitis B and D, talking about their disease status or fear of social stigma related to discussing positive test results. | Do not use when referring to visa, school, institutional, or employment specifically | 107 |
| **Quality of Life** | Use when emotions related to mood, mental health, quality of life are expressed (ie. feelings of depression, hopelessness or happiness, suicide, fear of death, optimism, stress, worry, concern) | Do not use when referring to stigma, discrimination, barriers, | 189 |
| **Treatment** | Use when asking about methods for treatment of hepatitis B or D, side effects, duration of treatment, liver transplant | Do not use when talking about medication/treatment access, financing etc. | 230 |
| **Clinical Trials** | Use when discussion involves questions about participating in clinical trials or questions about clinical trails | Do not use when talking about finding a doctor or medication access, or cost/financing treatment. | 82 |
| **Barriers** | Use when discussing barriers to treatment, medical care, general cost, poverty. | Do not use when referring to stigma, medication access or financing medication. | 22 |
| **Emotions fears and concerns** | Use when individual describes specific emotional implications of hepatitis B infection or fears associated with living with hepatitis B infection. | Do not use when referring to future treatment for hepatitis B infection | 243 |

**Interview Guide:**

**INTRODUCE TOPIC OF DISCUSSION:** Today, we will be discussing chronic hepatitis B (which I may refer to as CHB), with a particular focus on how it impacts you on a daily basis. We will be talking about your experiences throughout your journey with chronic hepatitis B and what you would like to see in the future. I would first like to start by finding out a little more about you.

1. Please briefly introduce yourself and tell me a little about yourself.
   - First name, age
   - How you spend your time? What things do you do?
   - Employment
   - Household setting and people in your life
2. Before we go any further, can I just double check what conditions you have been diagnosed with by a doctor?
   - **MODERATOR: IF PATIENT SAYS HEPATITIS PROBE AS TO WHAT TYPE OF HEPATITIS THEY HAVE BEEN DIAGNOSED WITH**

**MODERATOR - PLEASE LISTEN FOR HOW PATIENT DESCRIBES CHRONIC HEPATITIS B AND MIRROR THE LANGUAGE THROUGHOUT THE INTERVIEW**

**MODERATOR SAY: I would like to start at the beginning, and understand the experience of chronic hepatitis B from your point of view. First, please think back to before you were ever diagnosed with chronic hepatitis B. Could you briefly talk me through what led up to your diagnosis?**

1. Tell me how long ago you were diagnosed with chronic hepatitis B?
2. Do you know/ remember which symptoms or signs or event, if any, led to the diagnosis? How long before the formal diagnoses of chronic hepatitis B did the initial symptoms,signs, or events occur? What specifically prompted the visit to the doctor?
   - If you experienced symptoms, which symptoms did you first notice?
     - What did you think could be the cause of these symptoms before you got diagnosed with chronic hepatitis B?
     - What information did you look for or try to get hold of?
     - Where did you look for this information? Who did you ask?
     - How, if at all, were these symptoms affecting your daily life?
3. Who gave you the diagnosis of chronic hepatitis B?
   - What kind of doctor was this?
   - How did you come to see this doctor?
   - Do you remember how they described the condition to you?
   - Did they explain the causes of the condition?
   - What was your understanding of the condition at that point?
   - Do you remember the tests and examinations performed to confirm a diagnosis?
   - What questions did you have for your doctor about the condition?
4. Which type of doctor gave you the diagnosis?
   - Was it the first doctor you saw for your signs or symptoms? If not, who did you see first and how did you progress to the doctor who diagnosed you? Which other doctors or other healthcare professionals were involved when the diagnosis was made?
5. Do you remember what you were feeling when you understood you had chronic hepatitis B?
   - What concerns did you have, if any?
     - How did you address these concerns?
   - What hopes did you have, if any?
   - How did you imagine your life to be?
6. Before you were diagnosed with chronic hepatitis B had you heard of this condition? If you had heard of it, how did you hear of it and what specifically were you told or did you know or believe abou the condition before you were diagnosed?
7. **[ECO-MAPPING]** I would like to get an understanding of your world when it comes to being diagnosed with CHB.
   - Was there anyone with you at diagnosis? (PROBE on who/why..positive or negative associations)
   - Who did you tell about your condition when you found out? (PROBE on who/why…positive or negative associations)
   - Did anyone help you with your feelings or emotions around diagnosis? (PROBE)
   - Where did you find information about CHB? (PROBE for people, institutions, websites, etc)
   - Which health care professionals/hospital systems were involved? Who was most helpful or not at the time of diagnosis, or when you understood you had chronic hepatitis B?
8. Now we are going to use your imagination a little bit. If your chronic hepatitis B was an animal, what sort of animal would it be?
   - Explore the reasons behind the selection of animal
     - Positive characteristics
     - Negative characteristics
9. What is your understanding of the condition now? Are there areas that you are not sure about or still wish you understood better?
10. Please tell me about your life with chronic hepatitis B
    - How do you manage your day to day life with chronic hepatitis B?
11. **IF APPLICABLE:** What would you say is the biggest difference in your life between the time before your chronic hepatitis B diagnosis and now?
12. What impact has your diagnosis had on your family and those around you? What difference, if any, has it made to their lives?
13. How do you cope with being diagnosed with chronic hepatitis B?
    - How would you describe the impact of having chronic hepatitis B on your mood?
    - What effect, if any, has it had on your ability to do the activities that matter to you/ have the life that you desire to have?
    - What effect, if any, has it had on your family or those close to you and your relationship with them?
    - What effect, if any, has it had on your student/work life and your relationships with fellow students and/or workers?
    - What effect, if any, has it had on your social life and your relationships with your friends and/or other connections including your approach to new relationships
    - Are there any other impacts that chronic hepatitis B has had on your life that we have not yet discussed such as your diet or other areas?
14. Has your approach to living with chronic hepatitis B changed at all over time?
    - In what way and for what reasons?
    - Has the way you approach the condition emotionally changed over time?
    - Are you anxious or worried about this condition-why or why not?
15. **[ECO-MAPPING]** Thinking about all the people and systems we brought up before when it came to diagnosis, let’s think about the people and institutions around you as you navigate living with Chronic Heptatits B: Who and what are in your inner circle?
    - Is there anyone or a group of people you rely upon to cope with or deal with CHB day to day including any online support groups? (PROBE on who/why..positive or negative associations)
    - Who did you talk to about your condition, if anyone? Who else have you told about your condition since diagnoses? (PROBE on who/why…positive or negative associations)
    - Does anyone help you with your feelings or emotions around living with CHB? (PROBE)
    - Where do you find information about CHB as time goes on? (PROBE for people, institutions, websites/online resources, support groups, etc) And how frequently do you engage with these resources?
    - Which health care professionals/hospital systems have been involved since diagnoses? And who are involved now? Who are most helpful or not as time goes on? How well do you think the individuals you are involved with understand chronic hepatitis B? Do you feel that they are doing everything they can to help you with this condition?
    - Is anyone involved in your care now who is different from the time of diagnosis, or when you understood you had chronic hepatitis B?
16. Have you ever come across discrimination or felt any stigma attached to your diagnosis with chronic hepatitis B? Tell me more …
    - How do you cope with this particular aspect?
    - What impact has this had on your life? Has it impacted your family, friends, or your job?
17. Which symptoms, if any, do you experience overall due to chronic hepatitis B?
    - Which symptoms do you experience most often?
    - Which symptoms do you find the most bothersome?
    - How do you address these symptoms? And where/how did you learn about this approach to addressing these symptoms?
18. Have any of your physicians discussed the value of treatments, such as the outcomes or goals that you would see or achieve if you started treatment?
    - What specifically did they tell you would happen (or not happen) if you were treated for your chronic hepatitis B?
    - How believeable is/ was that?
19. Are you currently taking any prescription medication for your chronic hepatitis B?
    - **IF YES (ask the following and then go to Q24)** : If so, what are you currently prescribed?
      - Which physician type prescribed it?
      - For how long have you been prescribed it?
      - And what, if any, other treatments have you taken before this current treatment for your chronic hepatitis B since you were diagnosed?
        1. If you took other treatments, why were changes in your treatments made?
    - **IF NO: ASK Q22**
20. Have you ever taken any prescription medication for your chronic hepatitis B?
    - **IF YES (ask the following and then go to Q24):** If so, what have you been prescribed?
      - Which physician type prescribed it?
      - For how long did you take it?
      - For what reason did you stop taking it?
    - **IF NO: ASK Q23**
21. Do you know the reasons why you have never taken any prescription medication for your Chronic Hepatis B?
    - Has the subject of needing prescription medications for chronic hepatitis B ever been discussed with you?
    - If so, by whom?
      - What has been said?
      - What medications have been mentioned?
      - Why were these medications not prescribed to you?
    - If not, why do you think they have not discussed prescription medications with you?
    - What would need to happen for you to begin taking a prescription medication for your chronic Hepatitis B?

**ASK Q24-Q28 ONLY IF TREATED OR PREVIOUSLY TREATED:**

1. **(ECO-MAPPING)** Now thinking about making decisions around, and **discussing possible treatments** **for chronic hepatitis B**,?
   - Is there anyone involved in treatment decisions that wasn’t before? (PROBE on who/why..positive or negative associations)
   - What people or institutions (ie hospitals, insurance, access) is involved in treatment that you find you interact with most? (PROBE for positive/negative/neutral associations)
   - Does anyone help you with your feelings or emotions around treatment decisions? (PROBE)
   - Where do you find information about CHB treatment options? (PROBE for people, institutions, websites, etc)
   - Which health care professionals are involved? Who are most helpful or not? How well do you think they understand the CHB treatment options? Do you feel that they are doing everything they can to help you get the best treatment for your CHB?
2. How does being treated for chronic hepatitis B make you feel?
   - And how do the people around you feel about it?
   - Do you have any concerns about your treatment? Any hopes?
   - What have you been told about how long you need to stay on your treatment(s) for chronic hepatitis b? And how does this make you feel?

How do you describe your treatment for chronic hepatitis B? What is it doing for you and your condition? What are your hopes for this treatment?

1. To what extent do you feel your current treatment has been successful in treating your chronic hepatitis B?
   - What has it allowed you to achieve?
   - What has it prevented you from experiencing?
   - How has this impacted your life?
   - How do you think this has impacted the lives of those around you?
2. In general, considering your past and current treatments for chronic hepatitis B, have they met your expectations? Why or why not?
   - Do you think they have changed your signs or symptoms of chronic Hepatitis B? How so or not?
   - Do you think they have changed your long term impacts or outcomes from chronic Hepatitis B? How so or not?
   - Is there anything that still concerns you in relation to chronic Hepatitis B impacts or outcomes that you don’t think your current treatments are addressing?
3. Thinking of your current treatment(s), if you could change **one thing** about it to better suit your life, what would you change?
   - How would this impact your daily life?
   - How would it impact those around you?
   - What, if anything, would this allow you to do that you cannot do now?
